# Supplementary material for: Human occupation of northern India spans the Toba super-eruption ~74,000 years ago
Source: Nat Commun. 2020 Feb 25;11:961. doi: 10.1038/s41467-020-14668-4 (PMC7042215; doi:10.1038/s41467-020-14668-4)
Supplement: Supplementary file 1 — Supplementary Information [file 41467_2020_14668_MOESM1_ESM.pdf]

SUPPLEMENTARY INFORMATION

Human occupation of northern India spans the Toba super-eruption ~74,000 years ago

Clarkson et al.

Table of Contents:

Section 1: Sediment descriptions and artefact numbers.

Supplementary Table 1 | Stratigraphic description and artefact totals for each Dhaba trench.

Section 2: IRSL Dating

Supplementary Figure 1 | Typical IRSL and MET-pIRIR curves obtained for different stimulation temperatures.

Supplementary Figure 2 | Fading-corrected and –uncorrected D_e plotted against IR stimulation temperature for samples from Dhaba 1.

Supplementary Figure 3 | Fading-corrected and –uncorrected D_e plotted against IR stimulation temperature for samples from Dhaba 2.

Supplementary Figure 4 | Fading-corrected and –uncorrected D_e plotted against IR stimulation temperature for samples from Dhaba 3.

Supplementary Table 2: Summary of sampling depth, stratigraphic unit and the dosimetry data for the samples.

Supplementary Table 3: The single-aliquot regenerative-dose (SAR) protocol for multi-elevated-temperatures post-IR IRSL.

Section 3: Stone Artefacts

Supplementary Figure 5 | Levallois cores from Dhaba 1-3.

Supplementary Figure 6 | Microlithic blade cores from Dhaba 2 and 3.

Supplementary Figure 7 | Representative Levallois flakes and blades and retouched flakes from Dhaba 1 and 2.

Section 4: The Middle Son Valley Archaeological Sequence

Supplementary Discussion

Supplementary Figure 8 | Map of the Middle Son showing the location of sites mentioned in the text (after Jones and Pal 2009, fig. 1; Sharma and Clark 1983, fig. 1).

Section 5: Cryptotephra investigation

Supplementary Note 1

Supplementary Table 4 | Location of tephra shards at Dhaba 1.

Section 1: Sediment descriptions and artefact numbers.

Supplementary Table 1. Stratigraphic description and artefact totals for each Dhaba trench.

Stratigraphic Unit	Description	Munsell Colour	Number of Artefacts
	**Dhaba 1**		
**A**	Light brown compact sand of varying coarseness – frequent shale pebbles and granules. Rhizoliths and calcium carbonate nodules. Occasional shell. One animal burrow.	10YR 6/4	41
**B**	Light yellowish brown compact silt. Rhizoliths and calcium carbonate nodules. Occasional small rounded quartz pebbles and small angular shale.	10YR 6/4	24
**C**	Yellowish red compact silty clay. Occasional medium subangular cobbles of sandstone. Rhizoliths. One animal burrow.	5YR 5/6	19
**D**	Pale brown mottled with light grey brown loosely compact gravelly clay silt. Occasional small pockets of sand. Frequent calcium carbonate nodules, rhizoliths and granule-pebble sized shale clasts. Occasional small rounded quartz pebble.	10YR 6/3	249
**E**	Light yellowish brown compact clay silt. Occasional small pockets of sand. Extensive calcium carbonate nodules. High frequency of medium angular shale pebbles. Occasional quartzitic sandstone cobbles. Frequency and size of pebbles and cobbles increasing up-section. Stratum E occurs as a small lens within Stratum C.	10YR 6/4	34
**F**	Yellowish brown compact silt. Three animal burrows. Stratum F overlies G and H then becomes horizontally adjacent to Stratum G and H.	10YR 5/6	152
**G**	Yellowish brown clay-rich matrix-supported cobble to boulder gravel with angular quartzite clasts overlying bedrock.	10YR 5/4	196
**H**	Yellowish brown loose sandy clay. Frequent sand inclusions. Subangular shale.	10YR 5/4	146
**DHABA 2**			
**A**	Yellowish brown compact clay-rich silt –remobilised calcium carbonate nodules.	10YR 5/4	4
**B**	Yellowish brown compact clay-rich silt - calcium carbonate nodules.	10YR 5/4	5
**C**	Yellowish brown lens of calcium carbonate granules and pebbles.		8
**D**	Yellowish brown compact clay-rich silt - calcium carbonate nodules.	10YR 5/4	17
**E**	Light yellowish brown silt to sand.	10YR 5/4	1143
**F**	Yellowish brown medium sand – extends down to bedrock in places.	10YR 5/4	21

**G**	Yellowish brown sandy fine to medium sand with calcium carbonate nodules.	10YR 5/4	39
**H**	Brown pebbly clay.	10YR 5/3	641
**I**	Pale brown pebbly clay.	10YR 6/3	4
**DHABA 3**			
**A**	Light brownish gray, heavily oxidised soil with pebble lens	2.5Y 6/2	16
**B**	Light brownish gray sandy silty loosely compacted soil. Subvertical fractures. Low frequency of pebbles.	2.5Y 6/2	9
**C**	Light brownish grey clay rich sandy soil, very compact, pockets of granules and pebbles of quartzite and limestone. Overall low frequency of pebbles.	2.5Y 6/2	0
**D**	Light reddish brown silty very coarse sandy pebble gravel		23
**E**	Light reddish brown silty sandy small pebble gravel, occasional large cobbles		46
**F**	Light reddish brown silty sandy small pebble gravel, occasional large cobbles		396
**G**	Light reddish brown, very shale rich silty coarse sand		138
**H**	Light yellowish brown, very shale rich silty coarse Sand	2.5Y 6/3	155
**I**	Light yellowish brown sand, unsorted shale-rich silty coarse granule and contains few pebbles.	2.5 Y 6/3	365
**J**	Clast supported pebble infill (lens) that tapers to a very coarse sand-granule pebble pocket (to the right), and a pebble lag (to the left)	2.5 Y 6/3	247
**K**	Light yellowish brown, very unsorted shale-rich silty coarse granule gravel	2.5Y 6/3	197

Section 2: IRSL Dating

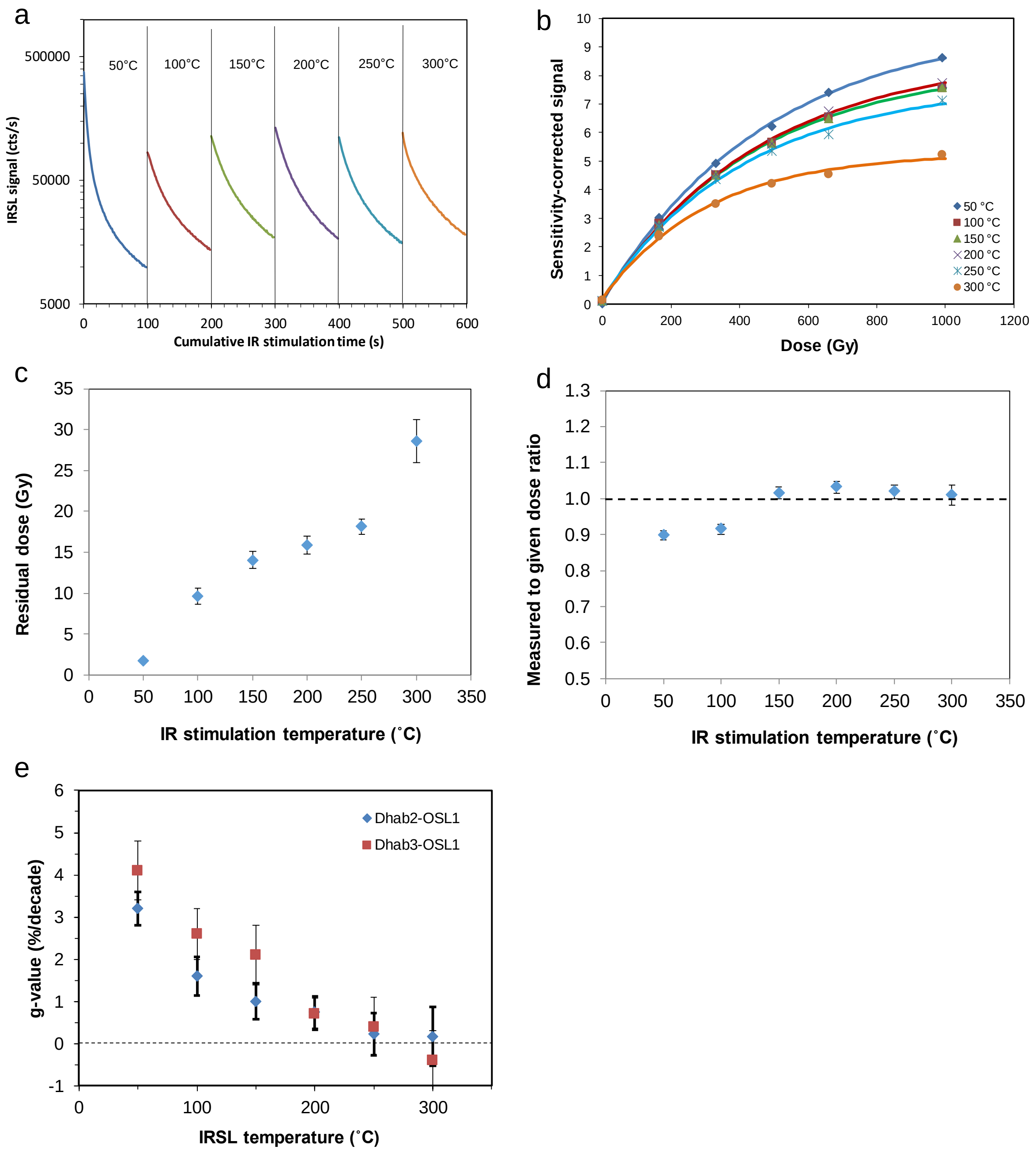

Supplementary Figure 1 | **a,** Typical IRSL and MET-pIRIR curves obtained for different stimulation temperature (temperatures are shown above each curve) from sample Dhaba1-OSL4. **b,** The dose response curves for the MET-pIRIR signals at different temperatures for sample Dhab1-OSL4. The data points were fitted using a single saturating exponential function (full lines).**c,** The residual doses of bleached sample (Dhab2-OSL4) plotted against the MET-pIRIR stimulation temperature. **d,** Dose recovery results (ratio of measured to given dose) for Dhab2-OSL4. The measured doses were corrected for residual dose shown in panel c. **e,** Anomalous fading rate (g-value) of the MET-pIRIR signals from samples Dhab2-OSL1 and Dhab3-OSL1 as a function of IR stimulation temperature. Error bars in all the panels represent 1 sigma uncertainty.

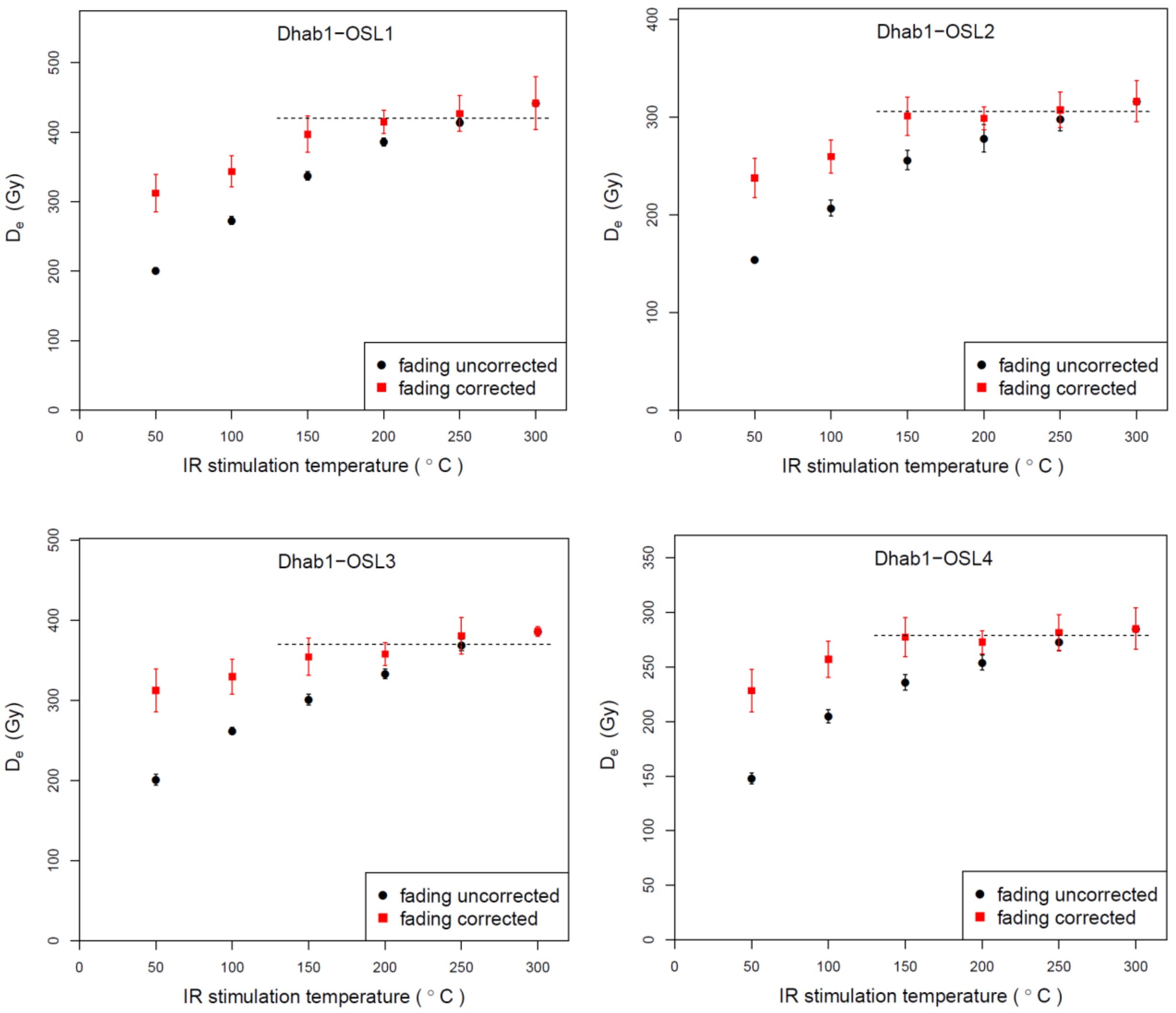

Supplementary Figure 2 | Fading-corrected and –uncorrected D_e plotted against IR stimulation temperature for samples from Dhaba 1. The stippled line in each plot shows the plateau D_e (i.e., mean of the fading corrected D_e values of those measured at 150˚C and above). Error bars represent 1 sigma uncertainty.

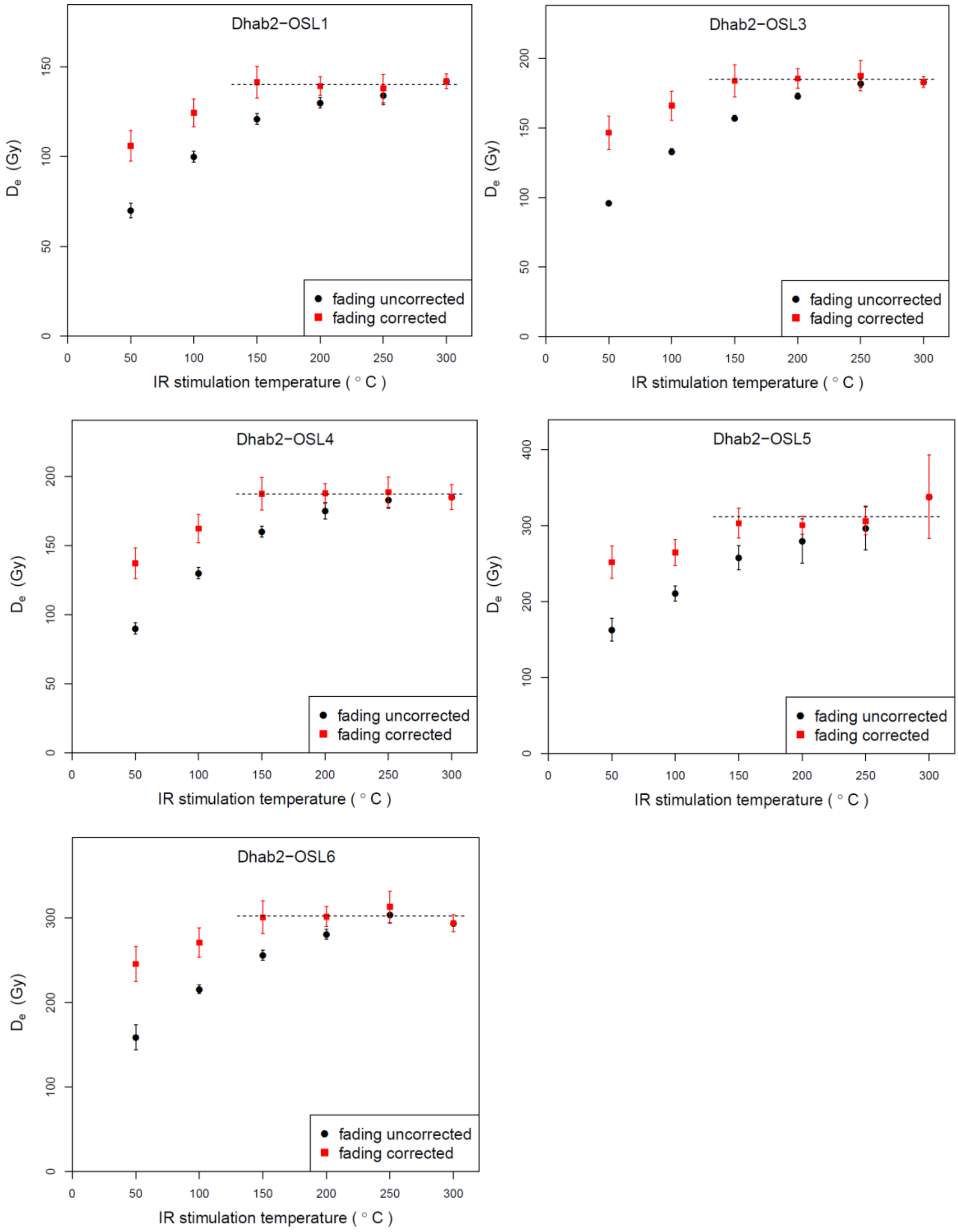

Supplementary Figure 3 | Fading-corrected and –uncorrected D_e plotted against IR stimulation temperature for samples from Dhaba 2. The stippled line in each plot shows the plateau D_e (i.e., mean of the fading corrected D_e values of those measured at 150˚C and above). Error bars represent 1 sigma uncertainty.

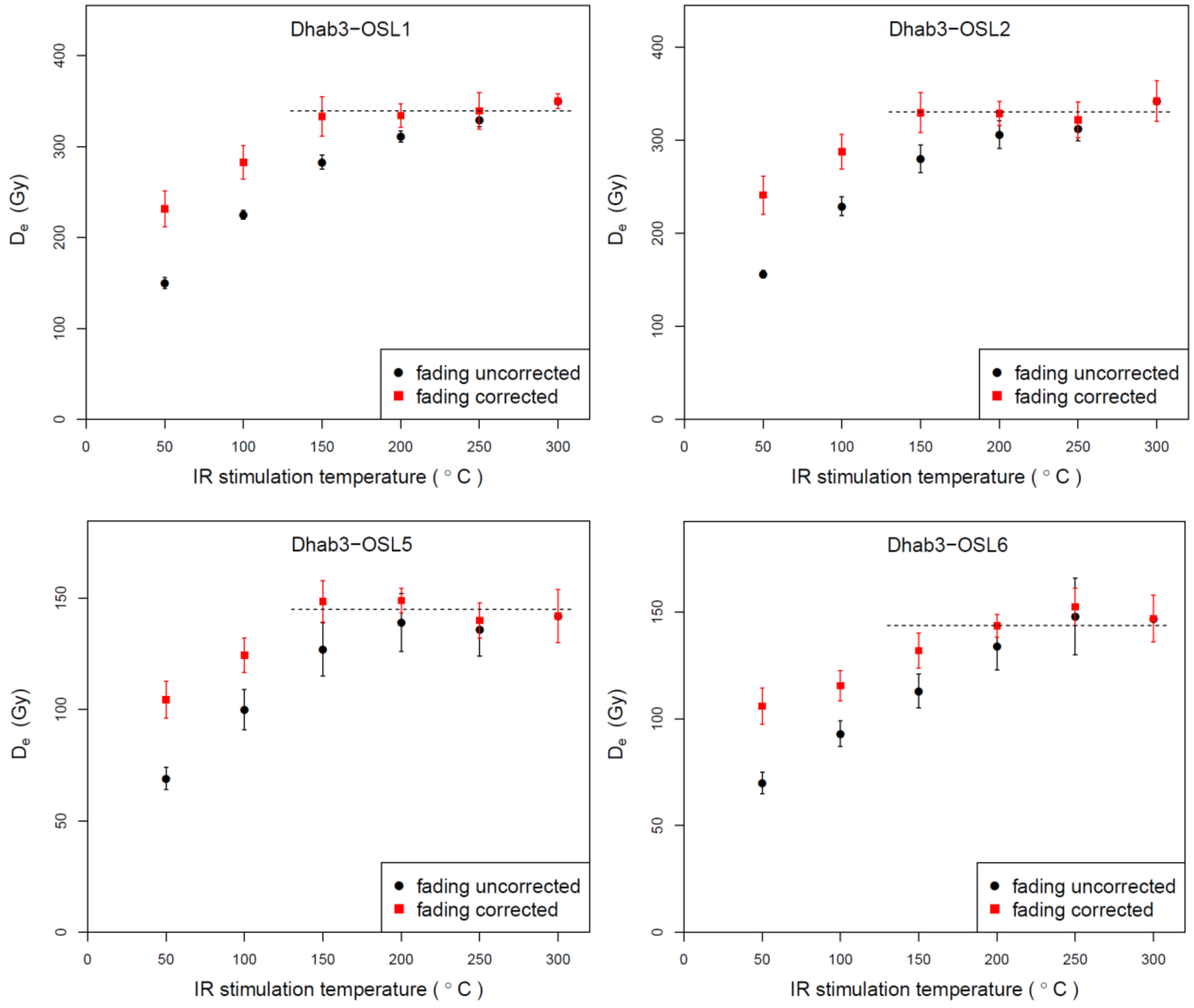

Supplementary Figure 4 | Fading-corrected and –uncorrected D_e plotted against IR stimulation temperature for samples from Dhaba 3. The stippled line in each plot shows the plateau D_e (i.e., mean of the fading corrected D_e values of those measured at 150˚C and above). Error bars represent 1 sigma uncertainty.

Supplementary Table 2: Summary of sampling depth, stratigraphic unit and the dosimetry data for the samples.

Sample	Depth (m)	Grain size (μm)	Ext. gamma dose rate (Gy/ka)	Ext. beta dose rate (Gy/ka)	Water content (%) [a]	Cosmic rays (Gy/ka)	Int. dose rate [b] (Gy/ka)	Total dose rate(Gy/ka)	Residual dose (Gy) [c]	D_e (Gy) [d]	Age(ka) [e]
**Dhaba 1**											
Dhab1-OSL4	6.8	125-180	1.34 ± 0.05	1.80 ± 0.07	7 ± 2 (4)	0.08	0.67 ± 0.08	3.90 ± 0.12	19 ± 1.1	254 ± 8	65.2 ± 3.1
Dhab1-OSL3	9.8	150-212	1.51 ± 0.06	2.11 ± 0.08	7 ± 2 (2)	0.06	0.79 ± 0.09	4.47 ± 0.13	13 ± 1.1	356 ± 7	79.6 ± 3.2
Dhab1-OSL2	10.8	150-212	1.41 ± 0.05	1.80 ± 0.07	7 ± 2 (5)	0.05	0.79 ± 0.09	4.05 ± 0.12	12 ± 1.1	286 ± 12	70.6 ± 3.9
Dhab1-OSL1	11.4	150-212	1.52 ± 0.06	2.81 ± 0.11	7 ± 2 (3)	0.05	0.79 ± 0.09	5.16 ± 0.15	11 ± 1	403 ± 5	78.0 ± 2.9
**Dhaba 2**											
Dhab2-OSL1	0.4	125-180	0.95 ± 0.04	1.50 ± 0.06	7 ± 2 (5)	0.19	0.67 ± 0.08	3.31 ± 0.11	11 ± 1	123 ± 5	37.1 ± 2.1
Dhab2-OSL3	2.7	150-212	1.08 ± 0.04	1.54 ± 0.06	7 ± 2 (2)	0.13	0.79 ± 0.09	3.54 ± 0.11	13.8 ± 1.2	168 ± 3	47.5 ± 2.0
Dhab2-OSL4	3.4	125-180	0.96 ± 0.04	1.31 ± 0.05	7 ± 2 (4)	0.12	0.67 ± 0.08	3.06 ± 0.10	18 ± 0.9	165 ± 6	53.9 ± 2.9
Dhab2-OSL5	5.6	150-212	1.62 ± 0.06	3.07 ± 0.12	7 ± 2 (2)	0.09	0.79 ± 0.09	5.58 ± 0.16	13.8 ± 1.2	283 ± 29	50.8 ± 5.5
Dhab2-OSL6	6.8	150-212	1.60 ± 0.07	2.85 ± 0.11	7 ± 2 (3)	0.08	0.79 ± 0.09	5.31 ± 0.15	12 ± 2	292 ± 10	55.0 ± 2.7
**Dhaba 3**											
Dhab3-OSL6	0.2	90-150	1.60 ± 0.09	2.64 ± 0.10	7 ± 2 (3)	0.21	0.54 ± 0.07	4.99 ± 0.16	13.8 ± 1.2	134 ± 18	26.9 ± 3.8
Dhab3-OSL5	0.6	90-125	1.75 ± 0.07	3.02 ± 0.12	7 ± 2 (2)	0.18	0.49 ± 0.05	5.44 ± 0.15	10 ± 2	126 ± 12	23.2 ± 2.4
Dhab3-OSL2	1.9	125-180	1.86 ± 0.07	3.45 ± 0.13	7 ± 2 (2)	0.15	0.67 ± 0.08	6.14 ± 0.17	13.8 ± 1.2	298 ± 13	48.6 ± 2.7
Dhab3-OSL1	2.7	90-180	1.78 ± 0.06	3.13 ± 0.12	7 ± 2 (3)	0.13	0.60 ± 0.10	5.64 ± 0.17	18 ± 0.9	311 ± 7	55.1 ± 2.4

Note:

[a] A water content of 7 ± 2 % was assumed for all samples, based on the measured (field) water content of each sample (shown in the brackets).

[b] The internal dose rate for K-feldspar used in age calculation was estimated by assuming K = 13 ± 1% and Rb = 400 ± 100 ppm.

[c] Residual doses of Dhab2-OSL3, Dhab2-OSL5, Dhab3-OSL2 and Dhab3-OSL6 were not measured, but are estimated based on the weighted mean of the residual doses measured for the other samples.

[d] D_es are estimated from the weighted mean of 6 aliquots of each sample. All the D_e values have been corrected for residual doses.

[e] Age uncertainties are expressed at 1σ and include a systematic error of 2% to allow for any possible bias associated with calibration of the laboratory beta source.

Supplementary Table 3: The single-aliquot regenerative-dose (SAR) protocol for multi-elevated-temperatures post-IR IRSL.

MET-pIRIR protocol		
Step	Treatment	Observed
1	Give regenerative dose, D_i[a]	
2	Preheat at 320°C for 60 s	
3	IRSL measurement at 50°C for 100 s	$L_{x(50)}$
4	IRSL measurement at 100°C for 100 s	$L_{x(100)}$
5	IRSL measurement at 150°C for 100 s	$L_{x(150)}$
6	IRSL measurement at 200°C for 100 s	$L_{x(200)}$
7	IRSL measurement at 250°C for 100 s	$L_{x(250)}$
8	IRSL measurement at 300°C for 100 s	$L_{x(300)}$
9	Give test dose, D_t	
10	Preheat at 320°C for 60 s	
11	IRSL measurement at 50°C for 100 s	$T_{x(50)}$
12	IRSL measurement at 100°C for 100 s	$T_{x(100)}$
13	IRSL measurement at 150°C for 100 s	$T_{x(150)}$
14	IRSL measurement at 200°C for 100 s	$T_{x(200)}$
15	IRSL measurement at 250°C for 100 s	$T_{x(250)}$
16	IRSL measurement at 300°C for 100 s	$T_{x(300)}$
17	IR bleaching at 325°C for 100 s	
18	Return to step 1	

[a]For the 'natural' sample, i= 0 and $D_0 = 0$. The whole sequence is repeated for several regenerative doses including a zero dose and a repeated dose.

Section 3: Stone Artefacts

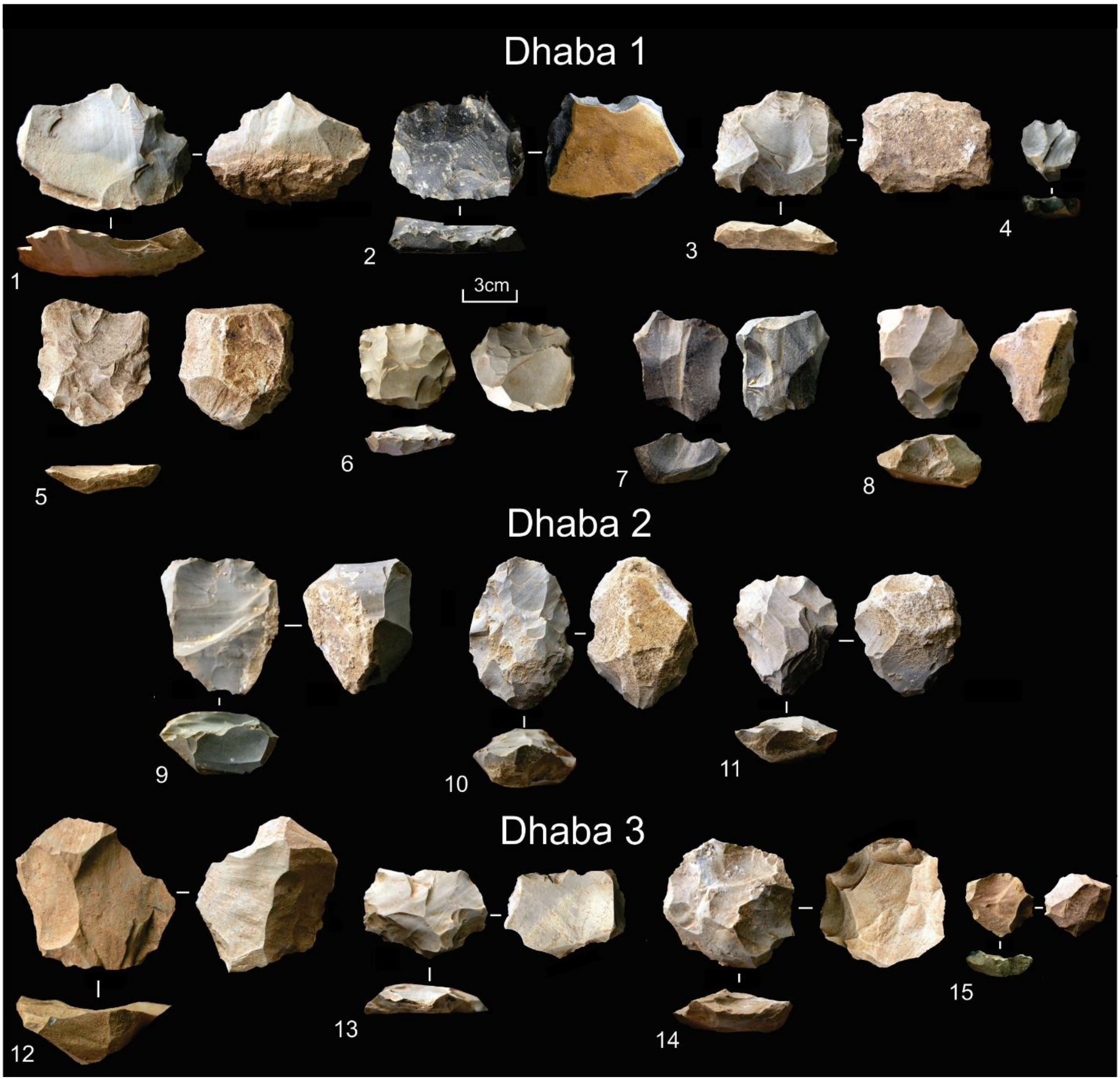

Supplementary Figure 5 | Levallois cores from Dhaba 1-3. 1. Unidirectional; 2. bidirectional; 3-6. centripetal; 7. unidirectional; 8. centripetal; 9. unidirectional; 10-15. centripetal.

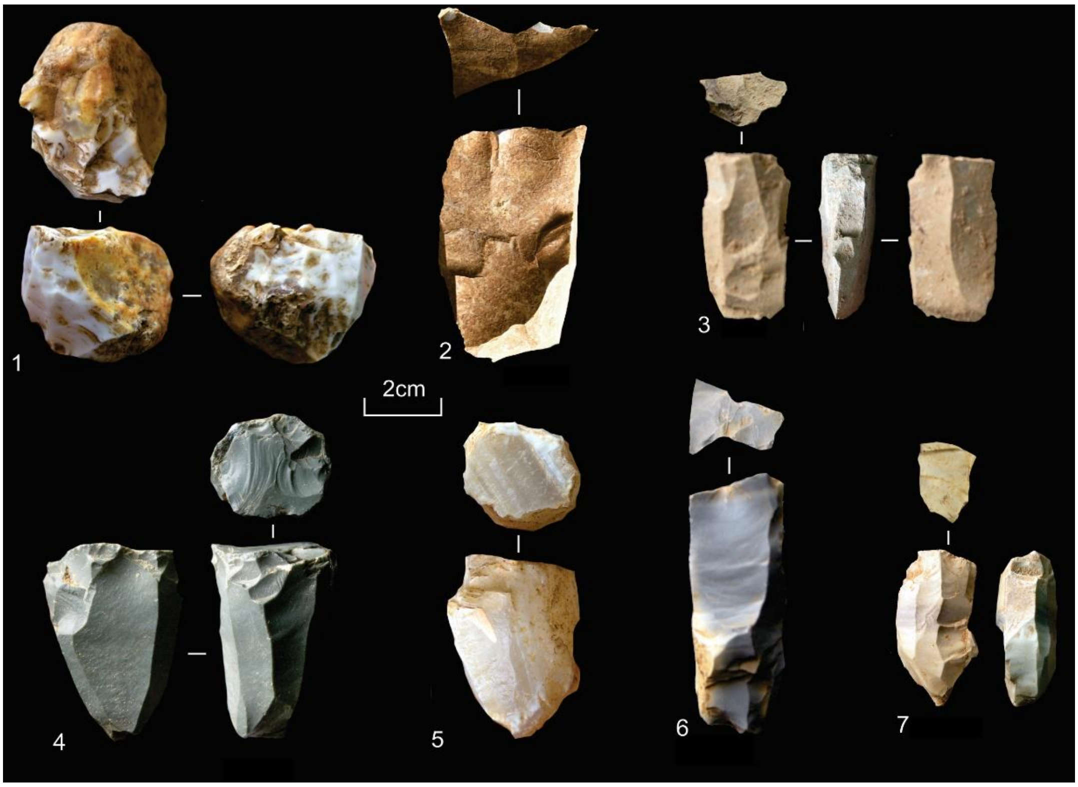

Supplementary Figure 6│Microlithic blade cores from Dhaba 2 and 3. 1. Agate pebble microblade core with three microblade scars; 2. bidirectional core; 3. limestone unidirectional microblade core; 4. bidirectional microblade core with faceted platform from the surface of the site; 5. agate unidirectional microblade core; 6-7. chert bidirectional microblade cores with faceted platforms.

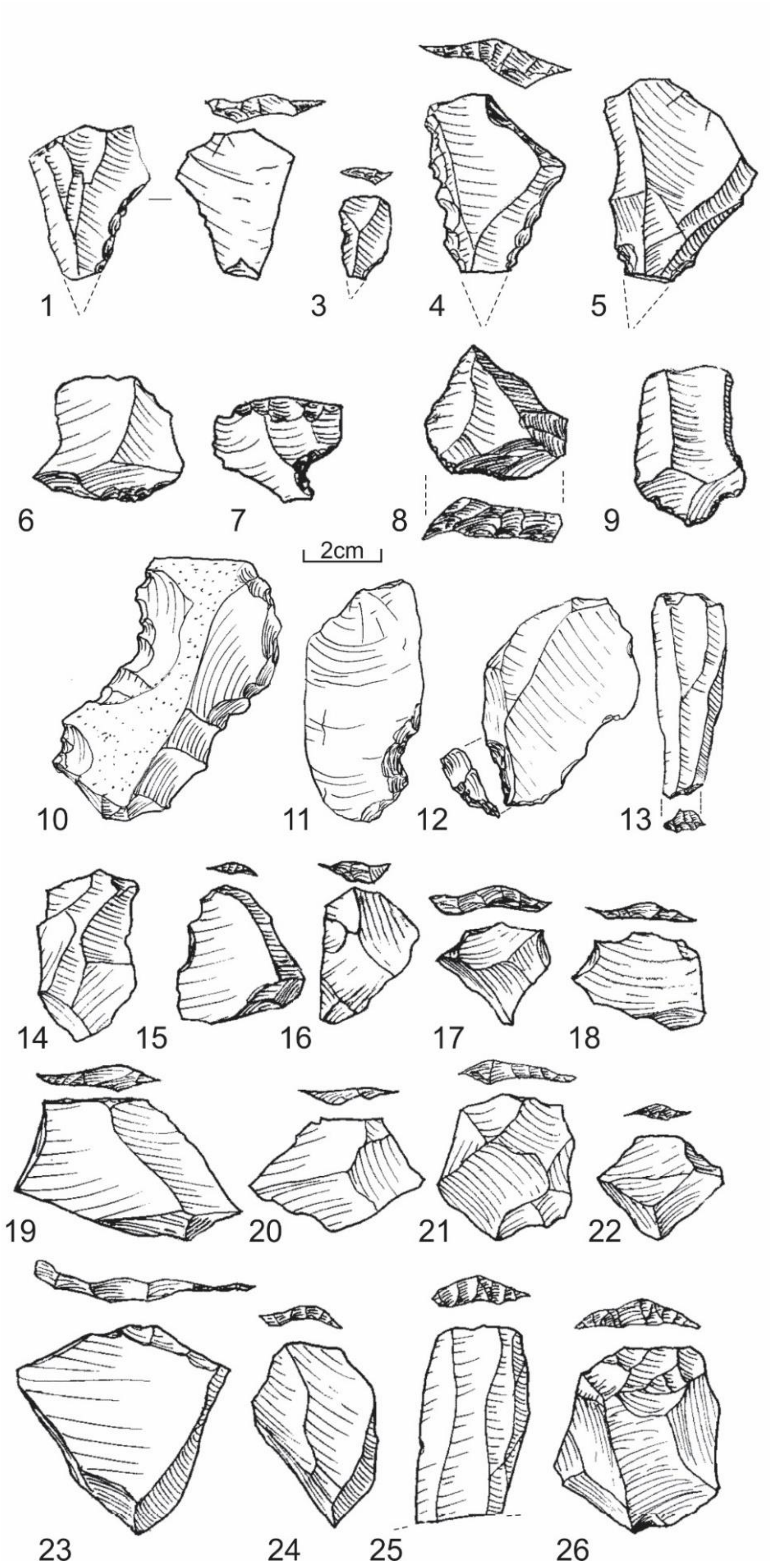

Supplementary Figure 7 | Representative Levallois flakes and blades and retouched flakes from Dhaba 1 and 2. 1–5. Levallois points; 6. end scraper; 7. notch; 8–9. end scrapers; 10. notched side scraper; 11. inverse notch; 12. side scraper; 13. end scraper on Levallois blade; 14–24. Levallois flakes; 25. broken Levallois blade; 26. Levallois flake.

Section 4 : The Middle Son Valley Archaeological Sequence

Supplementary Discussion

The Middle Son Valley is located in north-eastern Madhya Pradesh, approximately 100 km south of Allahabad and Varanasi. The Middle Son is bordered by the Kaimur Range to the north and the Baghelkhand plateau to the south, and shares a confluence with the Gopad River. Systematic archaeological research dates back to the early 1980s when Sharma and Clark[1] and collaborators carried out a multidisciplinary research programme in the area. They developed a sedimentological record for the region, between Patpara and the Gopad River, which detailed the sequence of deposition of late Quaternary geological formations as well as the likely relationship between these and broad cultural phases.

Initial geomorphological reconstructions of the alluvial deposits of the Middle Son identified four main geological formations; from oldest to youngest, these were termed Sihawal, Patpara, Baghor and Khetaunhi, with the Baghor formation divided into a lower coarse member and upper fine member[2]. Recent revisions of this sequence introduced a fifth formation, Khunteli, argued to post-date the accumulation of the Sihawal formation and pre-date that of the Patpara formation[3]. The introduction of this new formation, characterised by the presence of YTT and only described at two localities, is problematic for several reasons already outlined[4]. Recent OSL dates for sediments at Patpara, the type site of this formation, have produced ages of c.140 ka for initial deposition of Patpara formation sediments[5]; this is tens of thousands of years prior to the c.74 ka Toba eruption, indicating that the putative Khunteli formation cannot be older than the Patpara formation. In light of this evidence, and given the limited exposure of the Khunteli formation, it is perhaps prudent to discard it from the valley-wide geomorphological models. There is a more fundamental issue to raise: the modelled sequence of deposition in the valley ignores the considerable spatial variability in depositional processes both east to west along the river and to the north and south of its present-day course. In fact, overlapping chronometric dates for Sihawal and Patpara formation deposits indicate that these sediments accumulated synchronously rather than sequentially[6]. These complexities have consequences for how we understand, interpret and age the Middle Son valley's rich Palaeolithic cultural record.

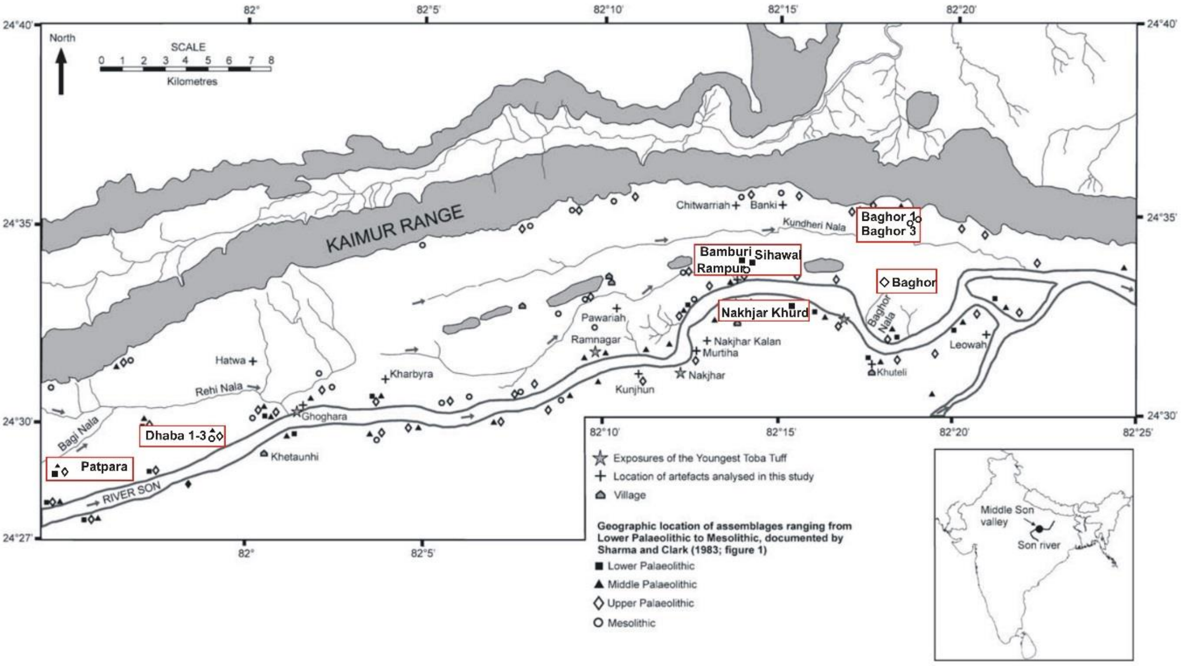

Supplementary Figure 8 | Map of the Middle Son showing the location of sites mentioned in the text [1,4].

Archaeological investigations in the valley by Sharma and Clark[1] documented a rich sequence of lithic artefacts recovered from excavations and surface collections, situated on the north and south banks of the Son River and at sites further north, towards the Kaimur foothills (Supplementary Figure 8). Artefacts span the Acheulean, through the Middle Palaeolithic and microlithic, and into the Neolithic periods[7-14]. This archaeological record was integrated into the valley's geomorphological record as follows: Lower Palaeolithic Acheulean technologies were associated with the Sihawal formation, Middle Palaeolithic with the Patpara formation, abraded Middle Palaeolithic and Upper Palaeolithic with the Baghor coarse member, Upper Palaeolithic and microlithic with the Baghor fine member, and microlithic and Neolithic pottery with the Khetaunhi formation[1,15].

Jones and Pal[4] conducted a technological and typological analysis of lithic artefacts deriving from these early investigations, assessing how hominin technologies changed through time from the late Acheulean, through the early and late Middle Palaeolithic, to the blade and bladelet (including microlithic) technologies of the later Palaeolithic. Central to their interpretations was a review and critique of all existing chronometric dates for the Middle Son, and the creation of a chronological model that hypothesizes the placement of sites and assemblages along a timeline that spans the Upper Pleistocene and the geological formations of the Son, and incorporates the Toba eruption (Fig. 3 in Jones and Pal[4]). Recent dates for pre-existing (Patpara) and new (Bamburi) archaeological sites[5] can now be used to update this model, alongside those presented for the first time in this paper.

Of great relevance to this study are the excavated localities of Patpara and Baghor, which preserve Late Acheulean to Middle Palaeolithic, and microlithic artefacts respectively. These pre-date and post-date Dhaba, dating to c.140 ka and younger at Patpara[5] and possibly terminal Pleistocene at Baghor, therefore leaving a large temporal gap in between. Other sites, which cover this gap and preserve late Middle Palaeolithic and early blade and bladelet technologies, are also highly relevant but evidence is limited[4]. Artefacts from Patpara, in the far west of the Middle Son study area, were assigned to the Lower and Middle Palaeolithic based on the morphology of the assemblages collected from surface scatters and excavations. The range of stone artefact types found at Patpara includes scrapers of varying edge modification and retouch intensity, notched artefacts, burins, handaxes, cleavers, backed artefacts and cores, including Levallois, discoidal and blade cores. Only two backed bladelets were recovered and are said to derive from overlying deposits that contain Upper Palaeolithic artefacts[16]. A technological analysis of recently excavated artefacts from Patpara reveals the *in situ* emergence of Levallois reduction methods as an adaptation of handaxe knapping[17]. The appearance of Upper Palaeolithic and microlithic technologies in tandem in the Middle Son is likely part of the same phenomenon documented in other regions of India[4,18] in that microlithic industries are not restricted to Mesolithic sites and there is not a linear chronological progression from Upper Palaeolithic to microlithic[19]. The excavated sites at the Baghor locality, Baghor 1, 2 and 3[7,9,14], are located in the northeast of the study area c.5 km north of the current river Son. They likely represent a late manifestation of the Upper Palaeolithic/microlithic in the area, yet the archaeological assemblages at these sites are either undated or poorly dated leaving us to guess at a terminal Pleistocene age. Artefacts from the Baghor sites derive from the fine member of the Baghor Formation[20] and the microlithic assemblage from Baghor 3 is the oldest of the three sites[9]. Artefacts from Baghor 3, manufactured on a range of high quality raw materials (e.g. chert, chalcedony, porcellanite), include blades, microblades, backed bladelets, burins and single platform bladelet cores, in addition to utilised red ochre[4,7].

While the artefact assemblages from Patpara and Baghor provide large sample sizes and consequentially a detailed record of past technologies and behaviour, there is a very large temporal gap between these excavated sites, in the order of c.100 kyr. In the Middle Son valley, this gap is covered by several very small collections of artefacts that are from unexcavated contexts, deriving instead from eroded surface and section exposures that are either undated or indirectly dated. Archaeologically, these small collections of artefacts are intriguing and are of particular relevance to this study. They are described in detail by Jones and Pal[4], who use evidence from initial reports[7,18,15], new fieldwork and artefact analysis[4,21]. To summarise, these collections derive from younger Patpara formation sediments and early

Baghor formation deposits, represented by the Baghor coarse member, proximal to the current river (and approximately equivalent to the Baghor loess deposits located more towards the Kaimur foothills). The late Patpara sediments have produced late Middle Palaeolithic artefacts, such as discoidal and Levallois cores, flakes and blades, and appears to mark a notable increase in the production of elongated blanks within a typically Middle Palaeolithic technological system. The Baghor deposits have also produced similar technologies (e.g. preferential Levallois flake cores and Levallois blade cores), both abraded and unabraded to suggest different scales of reworking, yet it is in this deposit that the first intensive production of artefacts typical of Upper Palaeolithic/microlithic industries is seen. This includes evidence of unidirectional and bidirectional blade and bladelet production and associated tools, such as multiple burins and backed points[4]. There are dates for Baghor coarse member deposits in the order of c.24–39 ka at Baghor Nala[3,12] but these are most likely minimum ages and no dates are directly associated with artefacts[4]. The evidence from the Dhaba locality is critical for addressing this elusive temporal gap in the Middle Son archaeological record. Deposits at Dhaba span the late Middle Palaeolithic to microlithic transition. Dhaba therefore greatly increases our knowledge of what has remained until now a poorly known period in the prehistory of the Middle Son.

Section 5: Cryptotephra investigation

Supplementary Note 1

A total of 32 discrete sediment samples, each weighing between 2 and 3 g, were collected at ~10 cm intervals throughout the sequence at Dhaba 1 and processed for cryptotephra analysis following the methods of Blockley et al.[22].

Samples were treated with dilute hydrochloric acid at 10% to remove carbonates, and then wet sieved to isolate sediments within the 25 – 80 µm grain size. Using the heavy liquid sodium polytungstate (SPT), the residues were further concentrated to contain only grains with a density in the 2.0 – 2.5 g/cm^3 range. The resultant residue was mounted onto glass slides using Canada balsam for shard identification under the microscope. The tephra shards appeared colourless and platy, with some open-vesicle structures.

Due to the low concentration of tephra glass shards (ranging from 0.5–1.2 shards per gram of sediment), it did not prove possible to geochemically fingerprint the tephra. While these shards are likely YTT, based upon the demonstrated presence of YTT within the Son River valley [4,21,23-25], chronological fit with the OSL ages of the sediment (this study) and correspondence of the visual appearance of the glass shards, we cannot rule out the possibility that these few shards are the result of human contamination from researchers working at both Dhaba 1 and the nearby site of Ghogara, where YTT deposits are abundant[24].

Supplementary Table 4. Location of tephra shards at Dhaba 1.

		Height above base section		
**OxT**	**Step**	**Base**	**Top**	**Tephra shard count / sample**
6729	4	0.08	0.1	1
6730	4	0.16	0.18	1
6731	4	0.25	0.27	0
6732	4	0.35	0.37	0
6733	4	0.46	0.48	0
6734	4	0.57	0.59	0
6735	4	0.67	0.69	0
6736	4	0.78	0.8	0
6737	4	0.87	0.89	0
6738	3	-0.01	0.01	0
6739	3	0.09	0.11	0
6740	3	0.18	0.2	0
6741	3	0.28	0.3	2
6742	2	0.03	0.05	0
6743	2	0.13	0.15	0
6744	2	0.23	0.25	0
6745	1	0.09	0.13	2
6746	1	0.19	0.23	0
6747	1	0.29	0.34	0
6748	1	0.38	0.43	0
6749	1	0.49	0.56	0
6750	2	0.33	0.35	0
6751	2	0.43	0.45	0
6752	1	0.54	0.56	0
6753	2	0.63	0.65	0
6754	1	0.62	0.64	0
6755	1	0.72	0.74	0
6756	1	0.92	0.94	0
6757	1	1.12	1.14	0
6758	1	1.32	1.34	0
6759	1	1.52	1.54	0
6760	1	1.75	1.74	0

Supplementary References

1. Sharma, G.R., & Clark, J.D. *Palaeoenvironments and prehistory in the Middle Son Valley*. Abinash Prakashan, Allahabad. (1983).
2. Williams, M.A.J., & Royce, K., Alluvial History of the Middle Son Valley, North Central India. In: Sharma, G.R. & Clark, J.D. (Eds.), Palaeoenvironments and Prehistory in the Middle Son Valley. Abinash Prakashan, Allahabad, pp. 9–22. (1983).
3. Williams, M.A.J., Pal, J.N., Jaiswal, M., & Singhvi, A.K., River response to Quaternary climatic fluctuations: evidence from the Son and Belan valleys, north-central India. *Quaternary Science Reviews* 25, 2619–2631. (2006).
4. Jones, S.C., & Pal, J.N., The Palaeolithic of the Middle Son valley, north-central India: changes in hominin lithic technology and behaviour during the Upper Pleistocene. *Journal of Anthropological Archaeology* 28, 323–341. (2009).
5. Haslam, M., Roberts, R.G., Shipton, C., Pal, J.N., Fenwick, J.L., Ditchfield, P., Boivin, N., Dubey, A.K., Gupta, M.C. & Petraglia, M., Haslam, M., et al. Late Acheulean hominins at the Marine Isotope Stage 6/5e transition in north-central India. *Quaternary Research* 75, 670–682. (2011).
6. Petraglia, M.D., Ditchfield, P., Jones, S., Korisettar, R., & Pal, J.N., The Toba volcanic super-eruption, environmental change, and hominin occupation history in India over the last 140,000 years. *Quaternary International 258*, 119–134. (2012).
7. Clark, J.D., & Dreiman, R., An occurrence with small blade technology in the Upper Member of the Baghor Formation at the Baghor III locality. In: Sharma, G.R. & Clark, J.D. (Eds.), *Palaeoenvironments and Prehistory in the Middle Son Valley*. Abinash Prakashan, Allahabad, pp. 197–208. (1983).
8. Kenoyer, J.M., & Pal, J.N., Report on the excavation and analysis of an Upper Acheulean assemblage from Sihawal II. In: Sharma, G.R. and Clark, J.D. (Eds.), *Palaeoenvironments and Prehistory in the Middle Son Valley*. Abinash Prakashan, Allahabad, pp. 23–38. (1983).
9. Kenoyer, J.M., Mandal, D., Misra, V.D., & Pal, J.N., Preliminary report on excavations at the Late Palaeolithic occupation site at Baghor I locality. In: Sharma, G.R. and Clark, J.D. (Eds.), 2006, *Palaeoenvironments and Prehistory in the Middle Son Valley*. Abinash Prakashan, Allahabad, pp. 117–142. (1983a).
10. Kenoyer, J.M., Clark, J.D., Pal, J.N., & Sharma, G.R., An Upper Palaeolithic shrine in India? *Antiquity* 57, 88–94. (1983b).
11. Misra, V.D., Mandal, D., Sinha, P., & Pal, J.N., An upper Palaeolithic collection from Rampur. In: Sharma, G.R., Clark, J.D. (Eds.), *Palaeoenvironments and Prehistory in the Middle Son Valley*. Abinash Prakashan, Allahabad, pp. 143–159. (1983).

12. Pal, J.N., Williams, M., Jaiswal, M., & Singhvi, A.K., Infrared stimulated luminescence ages for prehistoric cultures in the Son and Belan valleys, north central India. *Journal of Interdisciplinary Studies in History and Archaeology* 2(1), 51–62. (2005).
13. Sharma, G.R., & Clark, J.D., Palaeo-environments and prehistory in the Middle Son Valley, northern Madhya Pradesh. *Man and Environment* 6, 56–62. (1982).
14. Sussman, C., Blumenschine, R. J., Clark, J. D., & Misra, B. B., Preliminary report on excavations at the Mesolithic occupation site at Baghor II locality. In G. R. Sharma & J. D. Clark, (Eds.), *Palaeoenvironments and Prehistory in the Middle So0n Valley*. Abinash Prakashan, Allahabad, pp. 161–196. (1983).
15. Clark, J.D., & Williams, M.A.J., Paleoenvironments and prehistory in North Central India: a preliminary report. In: Jacobsen, J. (Ed.), *Studies in the Archaeology of India and Pakistan*. Aris and Phillips Ltd., Warminster, pp. 19–41. (1987).
16. Blumenschine, R.J., Brandt, S.A., & Clark, J.D., Excavations and analysis of Middle Palaeolithic artifacts from Patpara, Madhya Pradesh. In: Sharma, G.R. & Clark, J.D. (Eds.), *Palaeoenvironments and Prehistory in the Middle Son Valley*. Abinash Prakashan, Allahabad, pp. 39–99. (1983).
17. Shipton, C., Clarkson, C., Pal, J.N., Jones, S.C., Roberts, R.G., Harris, C., Gupta, M.C., Ditchfield, P.W. & Petraglia, M.D., Generativity, hierarchical action and recursion in the technology of the Acheulean to Middle Palaeolithic transition: a perspective from Patpara, the Son Valley, India. *Journal of Human Evolution* 65(2), 93-108. (2013).
18. Clark, J.D., & Sharma, G.R., A discussion of preliminary results and assessment of future research potential. In: Sharma, G.R. & Clark, J.D. (Eds.), *Palaeoenvironments and Prehistory in the Middle Son Valley.* Abinash Prakashan, Allahabad, pp. 261–280. (1983).
19. James, H.V.A., & Petraglia, M., Modern human origins and the evolution of behaviour in the later Pleistocene record of South Asia. *Current Anthropology* 46, S3–S27. (2005).
20. Williams, M.A.J., & Clarke, M.F., Quaternary geology and prehistoricenvironments in the Son and Belan valleys, North Central India. In: Wadia, S., Korisettar, R., Kale, V.S. (Eds.), *Quaternary Environments and Geoarchaeology of India*. Geological Society of India, Bangalore, pp. 282–308. (1995).
21. Jones, S.C., A human catastrophe? The impact of the ~74,000 year-old supervolcanic eruption of Toba on hominin populations in India. Unpublished PhD thesis, University of Cambridge, Cambridge. (2007).
22. Blockley, S.P.E., Pyne-O'Donnell, S.D.F., Lowe, J.J., Matthews, I.P., Stone, A., Pollard, A.M., Turney, C.S.M. & Molyneux, E.G., A new and less destructive laboratory procedure for the physical

separation of distal glass tephra shards from sediments. *Quaternary Science Reviews*, *24*(16-17), pp.1952-1960. (2005).

23. Westgate, J.A., Shane, P.A., Pearce, N.J., Perkins, W.T., Korisettar, R., Chesner, C.A., Williams, M.A. and Acharyya, S.K., All Toba tephra occurrences across Peninsular India belong to the 75,000 yr B.P. eruption. Quaternary Research 50, 107–112. (1998).
24. Gatti, E., Durant, A.J., Gibbard, P.L., & Oppenheimer, C., Youngest Toba Tuff in the Son Valley, India: a weak and discontinuous stratigraphic marker. *Quaternary Science Reviews* *30*(27-28), 3925–3934. (2011).
25. Smith, V.C., Pearce, N.J., Matthews, N.E., Westgate, J.A., Petraglia, M.D., Haslam, M., Lane, C.S., Korisettar, R. & Pal, J.N., Geochemical fingerprinting of the widespread Toba tephra using biotite compositions. *Quaternary International* 246, 97–104. (2011).
